# NICE shared decision making guidelines and mental health: challenges for research, practice and implementation

**DOI:** 10.1192/bjo.2021.987

**Published:** 2021-09-02

**Authors:** Yaara Zisman-Ilani, Marta Chmielowska, Lisa B. Dixon, Shulamit Ramon

**Affiliations:** Department of Social and Behavioral Sciences, College of Public Health, Temple University, USA; Centre for Outcomes Research and Effectiveness, University College London, UK; Division of Behavioral Health Services and Policies, New York State Psychiatric Institute, USA; Department of Allied Health, Midwifery and Social Work, University of Hertfordshire, UK

**Keywords:** Patients, shared decision making, serious mental illness, NICE, policy

## Abstract

The National Institute for Health and Care Excellence (NICE) initiated an ambitious effort to develop the first shared decision making guidelines. The purpose of this commentary is to identify three main concerns pertaining to the new published guidelines for shared decision making research, practice, implementation and cultural differences in mental health.

The National Institute for Health and Care Excellence (NICE) has a rich history of developing guidelines that tend to become ‘gold standards’ for healthcare practices and policies that are being adopted worldwide. The NICE formally adopted shared decision making (SDM) in 2015 as an important practice and goal in healthcare, and formed the SDM Collaborative, making NICE one of the first national health institutes globally to formally support SDM practice and research.^1^ SDM aims to facilitate patient-centred care and treatment adherence by promoting joint treatment decision-making between patients with chronic conditions and clinicians.^2^ In mental healthcare, SDM has been recommended for people with serious mental illness (SMI), given that self-determination, choice and autonomy are core aspects of recovery-oriented care.^3,4^

In December of 2018, NICE initiated an ambitious and important effort to develop the first guidelines for SDM in multiple physical and healthcare contexts. The hope is that the new guidelines, integrating ‘top-down’ theory and recommendations for patient engagement with ‘bottom-up’ patient and public feedback, will result in relevant, usable, acceptable and feasible guidance to facilitate SDM. The guidelines^1^ are based on five evidence documents focusing on the following: effectiveness of approaches and activities to increase engagement in SDM and the barriers and facilitators to engagement (Evidence Document A), interventions to support effective SDM (Evidence Document B), decision aids for people facing health treatment or screening decisions (Evidence Document C), risk communication (Evidence Document D) and effective approaches and activities to normalise SDM in the healthcare system (Evidence Document E).^5^ The purpose of this commentary is to share three main concerns pertaining to the implications of the new NICE guidelines on SDM in mental health (based on Evidence Documents A, B and E) that specifically affect the research, practice and implementation of SDM in mental health.

## Bias in the selection of qualified evidence for what is considered SDM in mental health

Our main concern is with bias in the presentation of what is considered representative evidence for effective SDM mental health research and practice, and, specifically, in SMI (e.g. schizophrenia, bipolar disorder, major depressive disorder), and its implications for the evolving research on SDM in mental health. The search criteria adopted by NICE exclude studies conducted with people in ‘situations in which people lack mental capacity to make their own decisions about healthcare at that time’.^1^ This is a problematic decision that excludes those considered to be ‘without capacity’ from engaging in the SDM process. It dismisses *a priori* evidence for rich SDM research with individuals with SMI,^3,6–8^ and enhances stigmatic beliefs about the ability of individuals with SMI and other concerns regarding capacity to engage in an SDM process, an assumption that is not yet evidence-based.^9,10^ As a result, the NICE recommendations for effectiveness approaches and activities to increase engagement in SDM (Evidence Document A) are based on only eight quantitative studies of SDM in mental health and ten qualitative studies. Of the included quantitative studies, only six^11–16^ are relevant to SMI and addressed SDM in community mental health settings (the others focused on primary care or patients with behavioural health issues^17^ or patients with dementia^18^). Only one quantitative study measuring recovery,^16^ a primary goal and outcome in mental health that is associated with SDM,^19–21^ was included. Of the included qualitative studies,^22–31^ only four^24–26,28^ involved the perspectives and attitudes of patients or individuals with mental health conditions on SDM (the rest addressed providers’ perspectives^22–25,^^27–31^), and most of these qualitative studies were valued by the NICE as having a high^22,27,28^ to moderate^23,24,26^ risk of bias.

SDM research in mental health has evolved rapidly in the past two decades,^3^ presenting one of the fastest growth curves in SDM research and practice. The first call for clinicians, patients and the scientific community to adopt SDM into mental health was in 2003, with a first overview of SDM in mental health introducing the concept and providing legitimacy of, and justification for, doing SDM in psychiatry.^32^ In 2010, a second review was published,^6^ identifying two German randomised controlled studies of SDM, one conducted in an acute in-patient psychiatric setting^33,34^ and the other in a community primary care setting.^35^ In 2017, a third review was published, including 31 unique studies, of which 20 were research articles evaluating SDM interventions.^3^ The latter demonstrated, for the first time, the heterogeneity of SDM tools and interventions for individuals with mental health conditions, including those with an SMI, and described a wide array of settings, research designs and various SDM in mental health outcomes, calling for an expansion of the narrow view of what can be considered SDM in mental health intervention and outcomes.^3^ Thus, the limited number of included SDM in mental health studies in the NICE guidelines is not representative of the richness of SDM research in mental health, its focus or its quality. Recommendations drawn on such a small poll of studies lag behind the emerging and developing field of research and practice, create a narrow representation of SDM in mental health, and lead to bias in future research and practice of SDM in mental health when designing and testing interventions, tools and measures.

## Bias in the selection of qualified outcomes and study designs for SDM in mental health studies

Compared with the limited number of included SDM in mental health studies, several important studies were excluded owing to a lack of high-quality evidence because of either a failure to present an objective primary outcome of SDM as defined by the NICE,^36–38^ the study design^38,39^ or the involvement of non-certified clinicians (e.g. peer workers) as the SDM providers.^40^

### What are the qualified outcomes of SDM in mental health?

The primary outcomes of SDM in mental health are often differ from outcomes in non-mental health studies (such as SDM use, knowledge and conflict level), and involve a focus on goals important to the field, such as empowerment, self-determination and recovery.^19,41,42^ Such outcomes are considered successful goals of SDM in mental health because of the many unique barriers to conducting SDM in mental health that exist at all levels (patients, clinicians, organisations and policy),^43,44^ making the mere act of engaging in an SDM process meaningful for empowering patients and promoting their recovery and self-determination.^42,45^ Therefore, in mental health research, SDM tools and interventions are often used to increase outcomes of empowerment, recovery, self-determination and hope, rather than to reduce decisional conflicts or increase knowledge^2,8,19,32,38^ – the ‘classical’ primary outcomes of interest common in non-mental-health SDM research.^46^ The lack of validated SDM measures uniquely developed to assess SDM in mental health is another factor contributing to the limited use of classical SDM outcomes in many studies of SDM in mental health. Since most existing measures for SDM in mental health were adapted or ‘borrowed’ from SDM studies in chronic physical illness (e.g. diabetes or cancer),^46^ the measured SDM output is often less relevant, meaningful and useful for assessing an SDM process in mental health.

### What research designs are appropriate for conducting SDM in mental health research?

Although the NICE inclusion criteria prioritised ‘randomised controlled trials (RCTs), well-designed quasi-experimental studies (quasi-RCTs), controlled clinical trials (CCTs), controlled before and after studies (CBAs) and interrupted time series analyses (ITS)’ (Evidence Document A), only randomised controlled trials or cluster randomised controlled trials were included in this review. We acknowledge the importance of randomised controlled trials as the ‘gold standard’ for causal evidence in health research. However, in health behavioural intervention studies, quasi-experimental designs are often the preferred alternative to generate strong causal evidence when blind randomisation is not feasible (e.g. because of ethical considerations, difficulty of randomising participants, difficulty randomising by location and small available sample size), and this is particularly true for studies of SDM in mental health.^47^ The nature of many SDM interventions in mental health studies,^3^ which focus on the humanistic live and interpersonal interaction between two participants (provider and patient), is particularly sensitive to risk of contamination and bias between the intervention and the control conditions because of the inherent processes that alter behaviour over time and may lead to type 2 error.^48^ Therefore, non-randomised quasi-research designs may be preferred and more common for SDM in mental health research. Excluding studies based on their non-RCT research design may contribute to bias in representing the evidence for effective SDM interventions in mental health.

Interestingly, NICE's focus on ‘primary objective criteria’ had led the committee to conclude that ‘as the primary outcome of “use of SDM” was not shown to be achieved, the secondary outcomes would not help inform the results of this review and therefore on the basis of the quantitative review, the committee were not able to recommend any interventions to increase engagement in SDM as effective’ and ‘based on the lack of robust quantitative evidence of the effectiveness of interventions, and the committee's lack of confidence in the quantitative data, the qualitative data was used as a guide for creating recommendations’ (Evidence Document A: The committee's discussion of the evidence). These conclusions provide further support for the bias selecting evidence based on narrow criteria addressing the type of primary outcomes and study design. At least in the mental health field, some of the studies excluded because of their design, outcome and focus could have proved invaluable when summarising the available SDM literature and developing recommendations.

## Bias in addressing cultural differences in SDM practice

The articles in Evidence Document B focused on SDM interventions. The search criteria adopted by the NICE exclude ‘non-English language papers’ studies conducted in ‘Non-OECD countries.’ As a result, only nine studies of SDM in mental health conducted in five Western countries, were included: USA,^14,17^ Germany,^49,50^ The Netherlands,^12,51^ Saudi Arabia^52^ and Japan.^16,53^ The majority of study populations included people of White or Northern European descent, or those belonging to a majority group in a given country. Thus, and based on the limited included SDM studies in mental health, there are no practical recommendations on the inclusion of culturally appropriate SDM recommendations for mental health practice. Facing a medical and ethical urgency to include Black, Asian and minority ethnic groups in clinical studies of SDM, randomised controlled trials have been recently cited as a potential source of structural racism and discrimination in healthcare research.^54–57^ Black, Asian and minority ethnic people are often more suspicious of participation in clinical controlled trials because of historic events (e.g. the Tuskegee syphilis study) and more current actions, including socioeconomic and healthcare system inequities.^57^ Expanding the definition of SDM in mental health to additional outcomes, decisions, populations and more naturalistic quasi-research design could have expanded the scope of the included articles, allowing better representation of cultural and ethnic diversity when conducting SDM research.^3^

To summarise, the upcoming NICE SDM guidelines represent a positive and important effort for patients, healthcare providers and families, policy makers and SDM researchers. We understand that in an effort to draw conclusions, there is a need to define clear boundaries between what is considered the gold standard in SDM evidence, tools and practice. Yet, we feel that with regard to SDM in mental health, the upcoming NICE recommendations are simplified, lag behind the emerging and developing field of research and practice, and create a narrow representation of SDM in mental health. The recommendations are also strongly biased toward Western countries and culture, as well as being White or belonging to a majority group.^58–61^ Considering the international impact of NICE guidelines on SDM practice, and since many studies of SDM in mental health fall outside of the NICE inclusion criteria, we worry that this narrow representation of SDM will become the gold standard, overlooking other effective research and interventions of SDM in mental health. For example, the NICE guidelines ignore the concept of shared advance decision making in mental health, SDM around decisions about advance care planning^62,63^ and the significant body of literature in mental health around the Joint Crisis Plan.^64,65^

We call on the NICE to revise their SDM recommendations for SDM studies, or at least to acknowledge that there exist other forms, designs and outcomes important for successful SDM in mental health. We hope that our call will foster an open and diverse discussion about the concept of SDM in mental health, the goals of SDM in mental health, define desired outcomes, support development of appropriate measures, acknowledge the needed diversity in research designs and address issues related to cultural relevancy.

## References

[1] National Institute for Health and Care Excellence (NICE). Shared Decision Making. NICE Guideline [NG197]. NICE, 2021 (https://www.nice.org.uk/guidance/ng197). 34339147

[2] Charles C, Gafni A, Whelan T. Shared decision-making in the medical encounter: what does it mean?(or it takes at least two to tango). Soc Sci Med 1997; 44(5): 681–92. 9032835 10.1016/s0277-9536(96)00221-3

[3] Zisman-Ilani Y, Barnett E, Harik J, Pavlo A, O'Connell M. Expanding the concept of shared decision making for mental health: a systematic and scoping review of interventions. Ment Heal Rev J 2017; 22(3): 191–213.

[4] Salyers MP, Zisman-Ilani Y. Shared decision-making and self-directed care. In The Palgrave Handbook of American Mental Health Policy (eds HH Goldman, RG Frank, JP Morrissey): 197–228. Springer International Publishing, 2020.

[5] National Institute for Health and Care Excellence (NICE). Shared Decision Making NICE Guideline [NG197] Evidence. NICE, 2021 (https://www.nice.org.uk/guidance/ng197/evidence).

[6] Duncan E, Best C, Hagen S. Shared decision making interventions for people with mental health conditions. Cochrane Database Syst Rev 2010; 1: CD007297. 10.1002/14651858.CD007297.pub2 PMC7209977 20091628

[7] Hauser K, Koerfer A, Kuhr K, Albus C, Herzig S, Matthes J. Outcome-relevant effects of shared decision making a systematic review. Dtsch Arztebl Int 2015; 112(40): 665–71. 26517594 10.3238/arztebl.2015.0665 PMC4640070

[8] Stovell D, Morrison AP, Panayiotou M, Hutton P. Shared treatment decision-making and empowerment-related outcomes in psychosis: systematic review and meta-analysis. Br J Psychiatry 2016; 209(1): 23–8. 27198483 10.1192/bjp.bp.114.158931

[9] Zisman-Ilani Y, Hurford I, Bowen A, Salzer M, Thomas EC. Shared Risk Taking: Shared Decision Making in Serious Mental Illness. Psychiatr Serv 2021; 72(4): 461–463. 33557595 10.1176/appi.ps.202000156

[10] Zisman-Ilani Y, Roth RM, Mistler LA. Time to Support Extensive Implementation of Shared Decision Making in Psychiatry. JAMA Psychiatr [Epub ahead of print] 18 Aug 2021. Available from: 10.1001/jamapsychiatry.2021.2247. 34406346

[11] Harris N, Lovell K, Day J, Roberts C. An evaluation of a medication management training programme for community mental health professionals; service user level outcomes. a cluster randomised controlled trial. Int J Nurs Stud 2009; 46(5): 645–52. 19070855 10.1016/j.ijnurstu.2008.10.010

[12] Metz MJ, Veerbeek MA, Twisk JWR, van der Feltz-Cornelis CM, de Beurs E, Beekman ATF. Shared decision-making in mental health care using routine outcome monitoring: results of a cluster randomised-controlled trial. Soc Psychiatry Psychiatr Epidemiol 2019; 54(2): 209–19. 30151651 10.1007/s00127-018-1589-8

[13] Metz M, Elfeddali I, Veerbeek M, de Beurs E, Beekman A, van der Feltz-Cornelis C. Effectiveness of a multi-facetted blended eHealth intervention during intake supporting patients and clinicians in shared decision making: a cluster randomised controlled trial in a specialist mental health outpatient setting. PLoS One 2018; 13(6): e0199795. 29944712 10.1371/journal.pone.0199795 PMC6019395

[14] Raue PJ, Schulberg HC, Bruce ML, Banerjee S, Artis A, Espejo M, Effectiveness of shared decision-making for elderly depressed minority primary care patients. Am J Geriatr Psychiatry 2019; 27(8): 883–93. 30967321 10.1016/j.jagp.2019.02.016 PMC6646064

[15] Woltmann EM, Wilkniss SM, Teachout A, McHugo GJ, Drake RE. Trial of an electronic decision support system to facilitate shared decision making in community mental health. Psychiatr Serv 2011; 62(1): 54–60. 21209300 10.1176/ps.62.1.pss6201_0054

[16] Yamaguchi S, Taneda A, Matsunaga A, Sasaki N, Mizuno M, Sawada Y, Efficacy of a peer-led, recovery-oriented shared decision-making system: a pilot randomized controlled trial. Psychiatr Serv 2017; 68(12): 1307–11. 28945186 10.1176/appi.ps.201600544

[17] Alegria M, Nakash O, Johnson K, Ault-Brutus A, Carson N, Fillbrunn M, Effectiveness of the DECIDE interventions on shared decision making and perceived quality of care in behavioral health with multicultural patients a randomized clinical trial. JAMA Psychiatry 2018; 75(4): 325–35. 29466533 10.1001/jamapsychiatry.2017.4585 PMC5875387

[18] Goossens B, Sevenants A, Declercq A, Van Audenhove C. Improving shared decision-making in advance care planning: implementation of a cluster randomized staff intervention in dementia care. Patient Educ Couns 2020; 103(4): 839–47. 31818522 10.1016/j.pec.2019.11.024

[19] Zisman-Ilani Y, Lysaker P, Hasson-Ohayon I. Shared risk taking: shared decision making in serious mental illness. Psychiatr Serv 2021; 72(4): 461–3. 33557595 10.1176/appi.ps.202000156

[20] Thornicroft G, Slade M. New trends in assessing the outcomes of mental health interventions. World Psychiatry 2014; 13(2): 118–24. 24890055 10.1002/wps.20114 PMC4102275

[21] Davidson L. Is “personal recovery” a useful measure of clinical outcome? Psychiatr Serv 2019; 70(12): 1079. 31787062 10.1176/appi.ps.701204

[22] Bradley E, Green D. Involved, inputting or informing: “shared” decision making in adult mental health care. Heal Expect 2018; 21(1): 192–200. 10.1111/hex.12601 PMC5750775 28779520

[23] Chong WW, Aslani P, Chen TF. Shared decision-making and interprofessional collaboration in mental healthcare: a qualitative study exploring perceptions of barriers and facilitators. J Interprof Care 2013; 27(5): 373–9. 23679672 10.3109/13561820.2013.785503

[24] Giacco D, Mavromara L, Gamblen J, Conneely M, Priebe S. Shared decision-making with involuntary hospital patients: a qualitative study of barriers and facilitators. BJPsych Open 2018; 4(3): 113–8. 29971154 10.1192/bjo.2018.6 PMC6020261

[25] Hamann J, Kohl S, McCabe R, Bühner M, Mendel R, Albus M, What can patients do to facilitate shared decision making? A qualitative study of patients with depression or schizophrenia and psychiatrists. Soc Psychiatry Psychiatr Epidemiol 2016; 51(4): 617–25. 26155899 10.1007/s00127-015-1089-z

[26] Lin CY, Renwick L, Lovell K. Patients’ perspectives on shared decision making in secondary mental healthcare in Taiwan: a qualitative study. Patient Educ Couns 2020; 103(12): 2565–70. 10.1016/j.pec.2020.05.030 32487469

[27] Mahone IH, Farrell SP, Hinton I, Johnson R, Moody D, Rifkin K, Participatory action research in public mental health and a school of nursing: qualitative findings from an academic-community partnership. J Particip Med 2011; 3: e-10. PMC3234528 22163075

[28] Mahone IH, Farrell S, Hinton I, Johnson R, Moody D, Rifkin K, Shared decision making in mental health treatment: qualitative findings from stakeholder focus groups. Arch Psychiatr Nurs 2011; 25(6): e27–36. 22114804 10.1016/j.apnu.2011.04.003 PMC3224341

[29] Patel SR, Schnall R, Little V, Lewis-Fernández R, Pincus HA. Primary care professional's perspectives on treatment decision making for depression with African Americans and Latinos in primary care practice. J Immigr Minor Heal 2014; 16(6): 1262–70. 10.1007/s10903-013-9903-8 PMC3981949 24104206

[30] Seale C, Chaplin R, Lelliott P, Quirk A. Sharing decisions in consultations involving anti-psychotic medication: a qualitative study of psychiatrists’ experiences. Soc Sci Med 2006; 62(11): 2861–73. 16343722 10.1016/j.socscimed.2005.11.002

[31] Shepherd A, Shorthouse O, Gask L. Consultant psychiatrists’ experiences of and attitudes towards shared decision making in antipsychotic prescribing, a qualitative study. BMC Psychiatry 2014; 14: 127. 24886121 10.1186/1471-244X-14-127 PMC4009071

[32] Hamann J, Leucht S, Kissling W. Shared decision making in psychiatry. Acta Psychiatr Scand 2003; 107(6): 403–9. 12752015 10.1034/j.1600-0447.2003.00130.x

[33] Hamann J, Langer B, Winkler V, Busch R, Cohen R, Leucht S, Shared decision making for in-patients with schizophrenia. Acta Psychiatr Scand 2006; 114(4): 265–73. 16968364 10.1111/j.1600-0447.2006.00798.x

[34] Hamann J, Cohen R, Leucht S, Busch R, Kissling W. Shared decision making and long-term outcome in schizophrenia treatment. J Clin Psychiatry 2007; 68(7): 992–7. 17685733 10.4088/jcp.v68n0703

[35] Loh A, Simon D, Wills CE, Kriston L, Niebling W, Härter M. The effects of a shared decision-making intervention in primary care of depression: a cluster-randomized controlled trial. Patient Educ Couns 2007; 67(3): 324–32. 17509808 10.1016/j.pec.2007.03.023

[36] Perestelo-Perez L, Rivero-Santana A, Sanchez-Afonso JA, Perez-Ramos J, Castellano-Fuentes CL, Sepucha K, Effectiveness of a decision aid for patients with depression: a randomized controlled trial. Heal Expect 2017; 20(5): 1096–105. 10.1111/hex.12553 PMC5600223 28295915

[37] Vigod S, Hussain-Shamsy N, Grigoriadis S, Howard LM, Metcalfe K, Oberlander TF, A patient decision aid for antidepressant use in pregnancy: study protocol for a randomized controlled trial. Trials 2016; 17: 110. 26923796 10.1186/s13063-016-1233-4 PMC4770694

[38] Zisman-Ilani Y, Roe D, Elwyn G, Kupermintz H, Patya N, Peleg I, Shared decision making for psychiatric rehabilitation services before discharge from psychiatric hospitals. Health Commun 2019; 34(6): 631–7. 29393685 10.1080/10410236.2018.1431018

[39] Lord K, Livingston G, Cooper C. A feasibility randomised controlled trial of the DECIDE intervention: dementia carers making informed decisions. BJPsych Open 2017; 3(1): 12–4. 28243460 10.1192/bjpo.bp.116.003509 PMC5288639

[40] Simmons MB, Batchelor S, Dimopoulos-Bick T, Howe D. The choice project: peer workers promoting shared decision making at a youth mental health service. Psychiatr Serv 2017; 68(8): 764–70. 28457208 10.1176/appi.ps.201600388

[41] Adams JR, Drake RE, Wolford GL. Shared decision-making preferences of people with severe mental illness. Psychiatr Serv 2007; 58(9): 1219. 17766569 10.1176/ps.2007.58.9.1219

[42] Drake RE, Deegan PE, Rapp C. The promise of shared decision making in mental health. Psychiatr Rehabil J 2010; 34(1): 7–13. 20615839 10.2975/34.1.2010.7.13

[43] Hamann J, Heres S. Adapting shared decision making for individuals with severe mental illness. Psychiatr Serv 2014; 65(12): 1483–6. 25756970 10.1176/appi.ps.201400307

[44] Slade M. Implementing shared decision making in routine mental health care. World Psychiatry 2017; 16(2): 146–53. 28498575 10.1002/wps.20412 PMC5428178

[45] Deegan PE, Drake RE. Shared decision making and medication management in the recovery process. Psychiatr Serv 2006; 57(11): 1636. 17085613 10.1176/ps.2006.57.11.1636

[46] Perestelo-Perez L, Rivero-Santana A, Alvarez-Perez Y, Zisman-Ilani Y, Kaminskiy E, Aguilar PS. Measurement issues of shared decision making in mental health: challenges and opportunities. Ment Heal Rev J 2017; 22(3): 214–32.

[47] Onken LS, Carroll KM, Shoham V, Cuthbert BN, Riddle M. Reenvisioning clinical science: Unifying the discipline to improve the public health. Clin Psychol Sci 2014; 2(1): 22–34. 25821658 10.1177/2167702613497932 PMC4374633

[48] Handley MA, Lyles CR, Mcculloch C, Cattamanchi A. Selecting and improving quasi-experimental designs in effectiveness and implementation research. Public Health 2018; 39: 5–25. 10.1146/annurev-publhealth-040617-014128 PMC8011057 29328873

[49] Hamann J, Mendel R, Meier A, Asani F, Pausch E, Leucht S, “How to speak to your psychiatrist”: shared decision-making training for inpatients with schizophrenia. Psychiatr Serv 2011; 62(10): 1218–21. 21969650 10.1176/ps.62.10.pss6210_1218

[50] Hamann J, Holzhüter F, Blakaj S, Becher S, Haller B, Landgrebe M, Implementing shared decision-making on acute psychiatric wards: a cluster-randomized trial with inpatients suffering from schizophrenia (SDM-PLUS). Epidemiol Psychiatr Sci 2020; 29: e137. 10.1017/S2045796020000505 PMC7303792 32539907

[51] Joosten EAG, de Weert GH, Sensky T, van der Staak CPF, de Jong CAJ. Effect of shared decision-making on therapeutic alliance in addiction health care. Patient Prefer Adherence 2008; 2: 277–85. 19920974 10.2147/ppa.s4149 PMC2770415

[52] Aljumah K, Hassali MA. Impact of pharmacist intervention on adherence and measurable patient outcomes among depressed patients: a randomised controlled study. BMC Psychiatry 2015; 15: 219. 26376830 10.1186/s12888-015-0605-8 PMC4574071

[53] Ishii M, Okumura Y, Sugiyama N, Hasegawa H, Noda T, Hirayasu Y, Feasibility and efficacy of shared decision making for first-admission schizophrenia: a randomized clinical trial. BMC Psychiatry 2017; 17: 52. 28166757 10.1186/s12888-017-1218-1 PMC5294770

[54] Courtright K. POINT: do randomized controlled trials ignore needed patient populations? Yes. Chest 2016; 149(5): 1128–30. 27157212 10.1016/j.chest.2016.01.029

[55] Hamel L, Penner L, Albrecht T, Heath E, Gwede C, Eggly S. Barriers to clinical trial enrollment in racial and ethnic minority patients with cancer. Cancer Control 2016; 23(4): 327–37. 27842322 10.1177/107327481602300404 PMC5131730

[56] Roe D, Jones N, Hasson-Ohayon I, Zisman-Ilani Y. Conceptualization and study of antipsychotic medication use: from adherence to patterns of use. Psychiatr Serv [Epub ahead of print] 15 Jun 2021. Available from: https://doi.org/101176/appi.ps202100006. 10.1176/appi.ps.202100006 34126781

[57] Scharff DP, Mathews KJ, Jackson P, Hoffsuemmer J, Martin E, Edwards D. More than Tuskegee: understanding mistrust about research participation. J Health Care Poor Underserved 2010; 21(3): 879. 20693733 10.1353/hpu.0.0323 PMC4354806

[58] Burkhard C, Cicek S, Barzilay R, Radhakrishnan R, Guloksuz S. Need for ethnic and population diversity in psychosis research. Schizophr Bull 2021; 47(4): 889–95. 33948664 10.1093/schbul/sbab048 PMC8266627

[59] Zisman-Ilani Y, Obeidat R, Fang L, Hsieh S, Berger Z. Shared decision making and patient-centered care in Israel, Jordan, and the United States: exploratory and comparative survey study of physician perceptions. JMIR Form Res 2020; 4(8): e18223. 32744509 10.2196/18223 PMC7432149

[60] Lewis-Fernández R, Raggio G, Gorritz M, Duan N, Marcus S, Cabassa LJ, GAP-REACH: a checklist to assess comprehensive reporting of race, ethnicity, and culture in psychiatric publications. J Nerv Ment Dis 2013; 201(10): 860–71. 24080673 10.1097/NMD.0b013e3182a5c184 PMC4324559

[61] Matthews EB, Savoy M, Paranjape A, Washington D, Hackney T, Galis D, Shared decision making in primary care based depression treatment: communication and decision-making preferences among an underserved patient population. Front Psychiatry 2021; 12: 1131. 10.3389/fpsyt.2021.681165 PMC8310927 34322040

[62] Swanson JW, Swartz MS, Elbogen EB, Van Dorn RA, Ferron J, Wagner HR, Facilitated psychiatric advance directives: a randomized trial of an intervention to foster advance treatment planning among persons with severe mental illness. Am J Psychiatry 2006; 163(11): 1943–51. 17074946 10.1176/appi.ajp.163.11.1943 PMC3747558

[63] de Jong M, Kamperman A, Oorschot M, Priebe S, Bramer W, van de Sande R, Interventions to reduce compulsory psychiatric admissions: a systematic review and meta-analysis. JAMA Psychiatry 2016; 73(7): 657–64. 27249180 10.1001/jamapsychiatry.2016.0501

[64] Thornicroft G, Farrelly S, Szmukler G, Birchwood M, Waheed W, Flach C, Clinical outcomes of Joint Crisis Plans to reduce compulsory treatment for people with psychosis: a randomised controlled trial. Lancet 2013; 381(9878): 1634–41. 23537606 10.1016/S0140-6736(13)60105-1

[65] Molyneaux E, Turner A, Candy B, Landau S, Johnson S, Lloyd-Evans B. Crisis-planning interventions for people with psychotic illness or bipolar disorder: systematic review and meta-analyses. BJPsych Open 2019; 5(4): e53. 10.1192/bjo.2019.28 PMC6582216 31530302

