# NICE shared decision making guidelines and mental health: challenges for research, practice and implementation – CORRIGENDUM

**DOI:** 10.1192/bjo.2021.1024

**Published:** 2021-11-16

**Authors:** Yaara Zisman-Ilani, Marta Chmielowska, Lisa B. Dixon, Shulamit Ramon

**Keywords:** Patients, shared decision making, serious mental illness, NICE, policy

The author and Publisher would like to correct the following references:

Reference 9 should be listed as:

Zisman-Ilani, Y, Lysaker, P., Hasson-Ohayon, I. Shared Risk Taking: Shared Decision Making in Serious Mental Illness. Psychiatr Serv 2021; 72(4): 461–463.

The Publisher apologises for this error.
